# Supplementary material for: Pathogenic and Genetic Diversity of Sclerotium rolfsii, the Causal Agent of Southern Blight of Common Bean in Uganda
Source: J Fungi (Basel). 2025 Dec 26;12(1):18. doi: 10.3390/jof12010018 (PMC12843155; doi:10.3390/jof12010018)
Supplement: Supplementary file 1 [file jof-12-00018-s001.zip › Table S5.pdf]

Table S5. The average severity of *S. rolfsii* strains from different agro-ecological zones in five experiments

S/no	Agro-ecology	Expt 1	Expt 2	Expt 3	Expt 4	Expt 5	Average
1	EH	30.6±4.0	NA	NA	NA	NA	30.6±2.2**
2	LVC	41.1±3.8	63.0±3.2**	66.7±13.1**	32.9±5.1	42.3±5.5	51.9±2.1
3	NMFS	57.5±3.7	55.6±3.1**	47.1±9.3	56.4±5.2	42.4±5.1	46.7±2.2
4	SWH	47.5±1.4	68.6±2.2	63.1±13.3	NA	NA	63.9±1.9**
5	WMFS	40.7±3.4	75.3±2.4	70.4±13.8	40.0±4.8	49.9±5.3	46.4±1.2
6	WNFS	39.3±3.5	NA	NA	52.5±3.4	56.1±5.4	49.1±2.4
7	TFZ	NA	36.7±2.1**	NA	NA	NA	36.7±2.0
8	TANZANIA	NA	NA	76.8±15**	NA	NA	76.8±1.9**

** = Significant at 95% confidence interval, NA = Isolates missing

EH = Eastern Highlands, LVC = Lake Victoria Crescent and Mbale Farmland, NMFS = Northern Mixed Farming System, SWH = South Western Highlands, TFZ = Teso Farming Zone, WMFS = Western Mixed Farming System, and WNFS = West Nile Mixed Farming System. ** = Significant at 0.05 confidence interval.
