# Supplementary material for: Pathogenic and Genetic Diversity of Sclerotium rolfsii, the Causal Agent of Southern Blight of Common Bean in Uganda
Source: J Fungi (Basel). 2025 Dec 26;12(1):18. doi: 10.3390/jof12010018 (PMC12843155; doi:10.3390/jof12010018)
Supplement: Supplementary file 1 [file jof-12-00018-s001.zip › Table S4.pdf]

Table S4. The proportion of ancestry of *S. rolfsii* isolates and their accession number at the Sequence Read Archive (SRA) data base

Isolate	Agro-ecology	Phi cluster 1	Phi cluster 2	Phi cluster 3	Phi cluster 4	Fhi cluster 5	Accession
SR11	NMFS	0.03586	0.281401	1.00E-04	1.00E-04	0.682539	SRX31254156
SR139	LVC	0.071883	1.00E-04	1.00E-04	0.927817	1.00E-04	SRX31254157
SR141	LVC	0.04007	0.304054	1.00E-04	1.00E-04	0.655676	SRX31254274
SR2	TFZ	0.346232	0.470204	1.00E-04	0.001811	0.181653	SRX31254285
SR200	EH	0.055375	1.00E-04	0.498469	0.443838	0.002218	SRX31254195
SR201	EH	0.052025	1.00E-04	0.506635	0.436819	0.004421	SRX31254206
SR203	LVC	0.070241	1.00E-04	0.002127	0.927432	1.00E-04	SRX31254290
SR205	LVC	0.352371	0.272038	1.00E-04	0.375391	1.00E-04	SRX31254301
SR207	LVC	0.030781	0.262159	1.00E-04	1.00E-04	0.70686	SRX31254312
SR208	LVC	0.081535	1.00E-04	0.01621	0.902055	1.00E-04	SRX31254250
SR209	LVC	0.326349	0.209431	0.46402	1.00E-04	1.00E-04	SRX31254158
SR22	SW	0.057281	1.00E-04	0.799238	0.143281	1.00E-04	SRX31254169
SR225	WNFS	0.074414	1.00E-04	0.008391	0.916996	1.00E-04	SRX31254180
SR228	WNFS	0.023985	0.469614	1.00E-04	1.00E-04	0.506201	SRX31254219
SR229	WNFS	0.074803	1.00E-04	0.040498	0.884499	1.00E-04	SRX31254230
SR23	SWH	0.069517	1.00E-04	0.007131	0.923152	1.00E-04	SRX31254241
SR235	WNFS	1.00E-04	0.901464	1.00E-04	1.00E-04	0.098236	SRX31254269
SR237	LVC	0.06685	1.00E-04	0.040806	0.892144	1.00E-04	SRX31254271
SR24	SWH	0.061082	1.00E-04	0.017603	0.921115	1.00E-04	SRX31254272
SR244	LVC	0.072015	1.00E-04	0.010965	0.91682	1.00E-04	SRX31254273
SR249	LVC	0.04927	1.00E-04	0.841487	0.109042	1.00E-04	SRX31254275
SR25	SW	0.450974	1.00E-04	0.429684	1.00E-04	0.119143	SRX31254276
SR250	LVC	0.373788	0.46986	1.00E-04	1.00E-04	0.156152	SRX31254277
SR252	LVC	0.022354	0.249983	1.00E-04	1.00E-04	0.727463	SRX31254278
SR256	LVC	0.9996	1.00E-04	1.00E-04	1.00E-04	1.00E-04	SRX31254279
SR26	SWH	0.9996	1.00E-04	1.00E-04	1.00E-04	1.00E-04	SRX31254280
SR279	WNFS	0.025096	0.269478	1.00E-04	1.00E-04	0.705226	SRX31254281
SR28	SWH	0.062324	1.00E-04	0.007286	0.93019	1.00E-04	SRX31254282
SR281	WNFS	0.000442	0.483268	1.00E-04	1.00E-04	0.51609	SRX31254283
SR282	WNFS	0.073	1.00E-04	0.015923	0.910878	1.00E-04	SRX31254284
SR283	WNFS	0.020183	0.327858	1.00E-04	1.00E-04	0.651759	SRX31254286
SR287	WMFS	0.055569	1.00E-04	0.500209	0.442892	0.00123	SRX31254287
SR29	SWH	0.04458	1.00E-04	0.808884	0.146335	1.00E-04	SRX31254288
SR290	WNFS	0.035169	0.313616	1.00E-04	1.00E-04	0.651015	SRX31254188
SR297	WMFS	0.08057	1.00E-04	0.016738	0.902493	1.00E-04	SRX31254189
SR30	SWH	0.060141	1.00E-04	0.004512	0.935148	1.00E-04	SRX31254190
SR302	WNFS	0.014399	0.332749	1.00E-04	1.00E-04	0.652652	SRX31254191
SR31	SWH	0.069022	1.00E-04	0.012978	0.9178	1.00E-04	SRX31254192
SR32	LVC	0.018748	0.328207	1.00E-04	1.00E-04	0.652845	SRX31254193
SR321	NMFS	0.352366	0.250201	1.00E-04	0.397233	1.00E-04	SRX31254194
SR323	NMFS	0.020797	0.306808	1.00E-04	1.00E-04	0.672195	SRX31254196
SR325	NMFS	0.315395	0.284328	0.400077	1.00E-04	1.00E-04	SRX31254197
SR33	NMFS	0.010129	0.628841	1.00E-04	1.00E-04	0.36083	SRX31254198

SR330	NMFS	0.078559	1.00E-04	0.011295	0.909946	1.00E-04	SRX31254199
SR332	NMFS	0.02456	0.266164	1.00E-04	1.00E-04	0.709076	SRX31254200
SR333	NMFS	0.315677	0.182021	0.502102	1.00E-04	1.00E-04	SRX31254201
SR334	NMFS	0.342353	0.217845	0.439602	1.00E-04	1.00E-04	SRX31254202
SR335	NMFS	0.306336	0.220012	0.473451	1.00E-04	1.00E-04	SRX31254203
SR336	NMFS	0.367619	0.179679	0.452501	1.00E-04	1.00E-04	SRX31254204
SR343	NMFS	0.077137	1.00E-04	0.014955	0.907708	1.00E-04	SRX31254205
SR35	NMFS	0.359763	0.257954	1.00E-04	0.382083	1.00E-04	SRX31254207
SR356	NMFS	0.070748	1.00E-04	0.017009	0.912043	1.00E-04	SRX31254208
SR37	WMFS	0.065676	1.00E-04	0.011922	0.922202	1.00E-04	SRX31254209
SR38	WMFS	0.024497	0.356308	1.00E-04	1.00E-04	0.618996	SRX31254210
SR4	TFZ	0.319862	0.334458	1.00E-04	0.34548	1.00E-04	SRX31254211
SR400	LVC	0.035736	1.00E-04	0.435948	0.52678	0.001437	SRX31254212
SR402	SWH	0.076041	1.00E-04	1.00E-04	0.923659	1.00E-04	SRX31254213
SR406	LVC	0.040477	1.00E-04	0.434203	0.522921	0.002299	SRX31254214
SR407	LVC	0.346797	0.200741	0.452262	1.00E-04	1.00E-04	SRX31254215
SR408	LVC	0.476043	1.00E-04	0.332012	1.00E-04	0.191745	SRX31254289
SR41	LVC	0.026735	0.360329	1.00E-04	1.00E-04	0.612736	SRX31254291
SR410	LVC	0.027799	1.00E-04	0.433839	0.532206	0.006057	SRX31254292
SR413	LVC	0.032708	0.287992	1.00E-04	1.00E-04	0.6791	SRX31254293
SR414	WMFS	0.444627	1.00E-04	0.382825	1.00E-04	0.172348	SRX31254294
SR415	LVC	0.028638	0.362513	1.00E-04	1.00E-04	0.608649	SRX31254295
SR417	TFZ	0.239858	0.3502	1.00E-04	0.409742	1.00E-04	SRX31254296
SR421	LVC	0.33335	0.243341	1.00E-04	0.423108	1.00E-04	SRX31254297
SR422	LVC	0.351135	0.203894	0.444771	1.00E-04	1.00E-04	SRX31254298
SR425	LVC	0.373707	0.268967	1.00E-04	0.357126	1.00E-04	SRX31254299
SR430	WMFS	0.011572	0.471035	1.00E-04	1.00E-04	0.517194	SRX31254300
SR433	WMFS	0.049709	1.00E-04	0.798648	0.151443	1.00E-04	SRX31254302
SR435	WMFS	0.072031	1.00E-04	0.00448	0.923289	1.00E-04	SRX31254303
SR436	WMFS	0.090646	1.00E-04	0.003433	0.905721	1.00E-04	SRX31254304
SR437	WMFS	0.066676	1.00E-04	1.00E-04	0.933025	1.00E-04	SRX31254305
SR438	WMFS	0.101494	1.00E-04	0.004594	0.893712	1.00E-04	SRX31254306
SR439	WMFS	0.067132	1.00E-04	0.00105	0.931617	1.00E-04	SRX31254307
SR443	WMFS	0.020982	0.335473	1.00E-04	1.00E-04	0.643345	SRX31254308
SR444	WMFS	0.056933	1.00E-04	0.789403	0.153464	1.00E-04	SRX31254309
SR446	WMFS	0.044518	1.00E-04	0.81829	0.136992	1.00E-04	SRX31254310
SR448	WMFS	0.041446	1.00E-04	0.825394	0.13296	1.00E-04	SRX31254311
SR449	WMFS	0.515154	1.00E-04	0.278272	1.00E-04	0.206374	SRX31254313
SR45	LVC	0.052282	1.00E-04	0.545288	0.391265	0.011065	SRX31254314
SR450	WMFS	0.036751	1.00E-04	0.403497	0.555902	0.00375	SRX31254315
SR454	WMFS	0.03517	0.278528	1.00E-04	1.00E-04	0.686102	SRX3125431
SR456	WMFS	0.025829	0.305316	1.00E-04	1.00E-04	0.668655	SRX31254244
SR458	LVC	0.334649	0.262219	0.402932	1.00E-04	1.00E-04	SRX31254245
SR459	LVC	0.30869	0.206698	0.484413	1.00E-04	1.00E-04	SRX31254246
SR46	LVC	0.02886	0.250073	1.00E-04	1.00E-04	0.720867	SRX31254247
SR460	LVC	0.9996	1.00E-04	1.00E-04	1.00E-04	1.00E-04	SRX31254248
SR462	LVC	0.340997	0.245412	1.00E-04	0.413391	1.00E-04	SRX31254249

SR464	LVC	0.9996	1.00E-04	1.00E-04	1.00E-04	1.00E-04	SRX31254251
SR466	LVC	0.338652	0.195406	1.00E-04	0.465743	1.00E-04	SRX31254252
SR468	LVC	0.070877	1.00E-04	0.024797	0.904126	1.00E-04	SRX31254253
SR47	LVC	0.044833	1.00E-04	0.428354	0.524229	0.002484	SRX31254254
SR471	LVC	0.340842	0.446048	0.21291	1.00E-04	1.00E-04	SRX31254255
SR472	LVC	0.015689	0.338059	1.00E-04	1.00E-04	0.646051	SRX31254256
SR474	LVC	0.057466	1.00E-04	0.466673	0.47474	0.001021	SRX31254257
SR475	LVC	0.974069	0.006671	1.00E-04	0.01906	1.00E-04	SRX31254258
SR476	LVC	0.061847	1.00E-04	0.461371	0.472684	0.003998	SRX31254259
SR477	LVC	0.448177	1.00E-04	0.288823	0.262801	1.00E-04	SRX31254260
SR478	LVC	0.040357	0.315043	1.00E-04	1.00E-04	0.6444	SRX31254159
SR48	LVC	0.519497	1.00E-04	0.127513	1.00E-04	0.35279	SRX31254160
SR481	LVC	0.9996	1.00E-04	1.00E-04	1.00E-04	1.00E-04	SRX31254161
SR482	LVC	0.05594	1.00E-04	0.473715	0.465214	0.005032	SRX31254162
SR484	LVC	0.333162	0.228082	1.00E-04	0.438556	1.00E-04	SRX31254163
SR485	LVC	0.032829	0.247788	1.00E-04	1.00E-04	0.719184	SRX31254164
SR487	LVC	0.323638	0.294827	0.381336	1.00E-04	1.00E-04	SRX31254165
SR488	LVC	0.373002	0.307414	0.319384	1.00E-04	1.00E-04	SRX31254166
SR491	LVC	0.353143	0.205193	1.00E-04	0.441464	1.00E-04	SRX31254167
SR492	LVC	0.031744	0.315839	1.00E-04	1.00E-04	0.652217	SRX31254168
SR493	LVC	0.029267	0.280614	1.00E-04	1.00E-04	0.689919	SRX31254170
SR494	LVC	0.50407	1.00E-04	0.283643	1.00E-04	0.212088	SRX31254171
SR495	LVC	0.059058	1.00E-04	0.444506	0.492572	0.003764	SRX31254172
SR497	LVC	0.035531	1.00E-04	0.445286	0.515193	0.003889	SRX31254173
SR498	NMFS	0.365247	0.259202	0.375351	1.00E-04	1.00E-04	SRX31254174
SR5	TFZ	0.012468	0.486199	1.00E-04	1.00E-04	0.501133	SRX31254175
SR500	NMFS	0.027555	0.263994	1.00E-04	1.00E-04	0.708252	SRX31254176
SR501	NMFS	0.042996	1.00E-04	0.823156	0.133647	1.00E-04	SRX31254177
SR502	NMFS	0.037584	0.335108	1.00E-04	1.00E-04	0.627107	SRX31254178
SR504	NMFS	0.042581	1.00E-04	0.431729	0.521565	0.004025	SRX31254179
SR505	LVC	0.031464	1.00E-04	0.412793	0.552169	0.003473	SRX31254181
SR506	LVC	0.295008	0.346639	0.358153	1.00E-04	1.00E-04	SRX31254182
SR508	NMFS	0.079204	1.00E-04	0.015627	0.90497	1.00E-04	SRX31254183
SR509	NMFS	1.00E-04	0.909008	1.00E-04	1.00E-04	0.090693	SRX31254184
SR510	WNFS	0.9996	1.00E-04	1.00E-04	1.00E-04	1.00E-04	SRX31254185
SR511	WNFS	1.00E-04	0.913019	1.00E-04	1.00E-04	0.086681	SRX31254186
SR512	WNFS	1.00E-04	0.906529	1.00E-04	1.00E-04	0.093171	SRX31254187
SR513	WNFS	1.00E-04	0.907332	1.00E-04	1.00E-04	0.092368	SRX31254216
SR514	WNFS	0.027125	0.329776	1.00E-04	1.00E-04	0.642899	SRX31254217
SR515	LVC	0.361774	1.00E-04	0.472631	1.00E-04	0.165396	SRX31254218
SR517	LVC	0.369065	1.00E-04	0.494827	1.00E-04	0.135908	SRX31254220
SR518	LVC	0.402652	1.00E-04	0.421393	1.00E-04	0.175755	SRX31254221
SR519	WMFS	0.06855	1.00E-04	0.006341	0.924909	1.00E-04	SRX31254222
SR52	LVC	0.374965	0.434931	0.189904	1.00E-04	1.00E-04	SRX31254223
SR522	WMFS	0.063925	1.00E-04	0.50774	0.426955	0.001279	SRX31254224
SR523	WMFS	0.028306	0.265503	1.00E-04	1.00E-04	0.705991	SRX31254225
SR524	WMFS	0.285729	0.181022	0.533049	1.00E-04	1.00E-04	SRX31254226

SR525	WMFS	0.346752	0.201302	0.451746	1.00E-04	1.00E-04	SRX31254227
SR527	WMFS	0.049669	0.321884	1.00E-04	1.00E-04	0.628247	SRX31254228
SR528	WMFS	0.047437	1.00E-04	0.820744	0.131619	1.00E-04	SRX31254229
SR529	WMFS	0.031069	0.265222	1.00E-04	1.00E-04	0.70351	SRX31254231
SR53	LVC	0.060099	1.00E-04	0.794578	0.145123	1.00E-04	SRX31254232
SR530	TANZANIA	0.458238	1.00E-04	0.136703	1.00E-04	0.404859	SRX31254233
SR531	TANZANIA	0.471793	1.00E-04	0.170809	1.00E-04	0.357198	SRX31254234
SR532	TANZANIA	0.068688	1.00E-04	0.517296	0.41159	0.002326	SRX31254235
SR533	TANZANIA	0.376508	0.268947	0.354344	1.00E-04	1.00E-04	SRX31254236
SR534	WMFS	0.039679	1.00E-04	0.506468	0.450558	0.003196	SRX31254237
SR55	LVC	0.025196	0.342753	1.00E-04	1.00E-04	0.631851	SRX31254238
SR56	LVC	0.9996	1.00E-04	1.00E-04	1.00E-04	1.00E-04	SRX31254239
SR57	LVC	0.373539	0.259745	1.00E-04	0.366516	1.00E-04	SRX31254240
SR59	LVC	0.356522	1.00E-04	0.483205	1.00E-04	0.160073	SRX31254242
SR6	LVC	0.041241	1.00E-04	0.432183	0.523277	0.003199	SRX31254243
SR63	LVC	0.241005	0.350044	0.408751	1.00E-04	1.00E-04	SRX31254261
SR65	LVC	0.031594	0.353218	1.00E-04	1.00E-04	0.614988	SRX31254262
SR67	LVC	0.031486	0.274948	1.00E-04	1.00E-04	0.693366	SRX31254263
SR70	LVC	0.016255	0.299567	1.00E-04	1.00E-04	0.683978	SRX31254264
SR74	LVC	0.361193	0.24699	1.00E-04	0.009864	0.381854	SRX31254265
SR77	LVC	0.482468	0.183834	0.040063	1.00E-04	0.293535	SRX31254266
SR8	NMFS	0.029057	0.207999	1.00E-04	1.00E-04	0.762744	SRX31254267
SR87	NMFS	0.048957	0.250422	1.00E-04	1.00E-04	0.70042	SRX31254268
SR9	NMFS	0.03483	1.00E-04	1.00E-04	1.00E-04	0.96487	SRX31254270

EH- Eastern Highlands, LVC- Lake Victoria Crescent and Mbale Farmland, NMFS- Northern Mixed Farming System, SWH- South Western Highlands, TFZ- Teso Farming Zone, WMFS- Western Mixed Farming System, WNFS- West Nile Mixed Farming System.
