# Supplementary material for: Pathogenic and Genetic Diversity of Sclerotium rolfsii, the Causal Agent of Southern Blight of Common Bean in Uganda
Source: J Fungi (Basel). 2025 Dec 26;12(1):18. doi: 10.3390/jof12010018 (PMC12843155; doi:10.3390/jof12010018)
Supplement: Supplementary file 1 [file jof-12-00018-s001.zip › Table S3.pdf]

Table S3. Mean and standard errors of morphological and pathogenicity characteristics of *S. rolfsii* strains from five genetically distinct clusters

Group	Strains	DSI (%)	Growth rate (cm/day)	Average Sclerotia number
1	50	52.8±2.4 ab	2.43±0.05 b	25.2 b
2	8	50.6±2.3 ac	2.68±0.05 ab	15.8 b
3	32	47.4±2.3 b	2..62±0.05 ab	35.8 b
4	13	56.0±2.2 ab	2.57±0.05 ab	31.8 b
5	58	57.6±2.4 a	2.66±0.05 a	115 a
