# Supplementary material for: Pathogenic and Genetic Diversity of Sclerotium rolfsii, the Causal Agent of Southern Blight of Common Bean in Uganda
Source: J Fungi (Basel). 2025 Dec 26;12(1):18. doi: 10.3390/jof12010018 (PMC12843155; doi:10.3390/jof12010018)
Supplement: Supplementary file 1 [file jof-12-00018-s001.zip › Table S2.pdf]

Table S2. Growth rate (cm/day), number of sclerotia produced by *S. rolfsii* strains, and their genetic cluster. The number of sclerotia was characterised as: 0- none, low- 1 to 49, Medium- 50 to 99, and high- 100 and above. Strains having NA were absent during genotyping

S/no	Strain	Year	District	Agro-ecology	Growth (cm/day)	SE	Sclerotia number	Group	Genetic Cluster
1	SR468	2021	Sironko	LVC	3.67	0	290	High	5
2	SR6	2013	Apac	NMFS	3.63	0.01	0	None	5
3	SR505	2020	Hoima	WMFS	3.6	0.05	99	Medium	5
4	SR495	2021	Sironko	LVC	3.57	0	0	None	5
5	SR495	2021	Sironko	LVC	3.57	0.07	0	None	5
6	SR462	2021	Sironko	LVC	3.5	0.13	132	High	3
7	SR462	2021	Sironko	LVC	3.5	0.04	132	High	3
8	SR517	2021	Sironko	LVC	3.47	0.01	0	None	NA
9	SR517	2021	Hoima	WMFS	3.47	0.01	0	Low	NA
10	SR477	2021	Sironko	LVC	3.43	0.01	56	Medium	5
11	SR464	2021	Sironko	LVC	3.33	0.11	0	None	2
12	SR446	2021	Hoima	WMFS	3.33	0.04	21	Low	5
13	SR446	2021	Hoima	WMFS	3.33	0.01	21	Low	5
14	SR205	2013	Jinja	LVC	3.23	0.08	171	Medium	5
15	SR472	2021	Sironko	LVC	3.223	0.04	0	None	NA
16	SR438	2021	Hoima	WMFS	3.2	0.05	101	High	5
17	SR438	2021	Hoima	NMFS	3.2	0.06	101	Medium	5
18	SR32	2013	Kamuli	LVC	3.2	0.13	4	Low	1
19	SR508	2021	Arua	WNFS	3.18	0.03	115.7	medium	5
20	SR250	2013	Rakai	LVC	3.13	0.01	35	Low	1
21	SR482	2021	Sironko	LVC	3.13	0.03	306.3	High	5
22	SR250	2013	Rakai	LVC	3.13	0	35	Low	1
23	SR450	2021	Hoima	WMFS	3.1	0	480.7	High	NA
24	SR46	2013	Luwero	LVC	3.1	0.03	0	None	1
25	SR487	2021	Sironko	LVC	3.1	0.03	23	Low	3
26	SR65	2013	Mukono	LVC	3.1	0.11	0	None	1
27	SR487	2021	Sironko	LVC	3.1	0.01	23	Low	3
28	SR63	2013	Mukono	LVC	3.1	0.05	0	None	3
29	SR53	2013	Mbale	LVC	3.1	0.07	0	None	5
30	SR449	2021	Hoima	WMFS	3.08	0.04	80	Medium	4
31	SR282	2013	WMFS	Unk	3.07	0.07	7.3	Low	5
32	SR203	2013	Kamwenge	WMFS	3	0.04	529	High	5
33	SR400	2013	Mbale	LVC	3	0.03	0	None	5
34	SR415	2013	Mukono	LVC	3	0.02	31.35	None	1
35	SR74	2013	Nakaseke	LVC	3	0.02	0	None	1
36	SR491	2021	Sironko	LVC	3	0.06	26	Low	3
37	SR45	2013	Luwero	LVC	3	0.08	76	medium	5
38	SR74	2013	Nakaseke	LVC	3	0.07	0	None	1
39	SR421	2013	Luwero	LVC	3	0.17	29	Low	3
40	SR74	2013	Nakaseke	LVC	3	0.03	0	None	1
41	SR458	2021	Sironko	LVC	3	0.02	60	Medium	NA
42	SR475	2021	Sironko	LVC	2.97	0.13	47	Low	2

43	SR534	2013	WMFS	LVC	2.92	0.11	0	None	NA
44	SR534	2013	Mbale	LVC	2.92	0.05	0	None	NA
45	SR425	2013	Sironko	LVC	2.91	0.01	0	None	3
46	SR407	2013	Luwero	LVC	2.91	0.02	0	None	3
47	SR67	2013	Mukono	LVC	2.9	0.02	0	None	1
48	SR530	2021	Bukoba	Tanzania	2.9	0.05	0	None	4
49	SR530	2021	Bukoba	Tanzania	2.9	0.08	0	None	4
50	SR290	2013	Kamwenge	WMFS	2.9	0.04	54	Medium	1
51	SR484	2021	Sironko	LVC	2.9	0.08	217.3	High	3
52	SR139	2013	Sironko	TFZ	2.9	0.06	1	Low	5
53	SR408	2013	Nakaseke	LVC	2.9	0.04	0	None	4
54	SR52	2013	Luwero	LVC	2.9	0.07	0	None	3
55	SR47	2013	Luwero	LVC	2.9	0.04	44.35	Low	5
56	SR56	2013	Mbale	LVC	2.9	0.13	0	None	2
57	SR252	2013	Masindi	WMFS	2.88	0	35	Low	1
58	SR501	2020	Oyam	NMFS	2.87	0	22.3	Low	5
59	SR256	2013	WMFS	WMFS	2.85	0.17	9	Low	2
60	SR494	2021	Sironko	LVC	2.85	0.07	0	None	4
61	SR532	2013	Mbale	LVC	2.85	0.04	0	None	NA
62	SR237	2013	Lwengo	LVC	2.83	0.04	256	High	5
63	SR522	2021	Hoima	WMFS	2.83	0.03	65	Medium	5
64	SR504	2020	Oyam	NMFS	2.82	0.05	248	High	5
65	SR244	2013	WMFS	WMFS	2.8	0.05	44	Low	5
66	SR435	2021	Hoima	WMFS	2.8	0.08	65	Medium	5
67	SR281	2013	Kabarole	SWH	2.8	0.04	476	High	5
68	SR77	2013	Nakaseke	LVC	2.8	0.03	0	None	4
69	SR413	2013	Kayunga	LVC	2.8	0.02	10.01	Low	1
70	SR45	2013	Luwero	LVC	2.8	0.07	76.01	medium	5
71	SR410	2013	Mbale	LVC	2.8	0.05	72.01	medium	5
72	SR38	2013	Kyenjojo	WMFS	2.8	0.08	0.3	Low	1
73	SR456	2021	Sironko	LVC	2.78	0.02	0	None	1
74	SR433	2021	Hoima	WMFS	2.77	0.02	64	Medium	5
75	SR283	2013	Kyenjojo	WMFS	2.77	0.04	8	Low	1
76	SR249	2013	WMFS	WMFS	2.76	0.04	74	Medium	5
77	SR481	2021	Sironko	LVC	2.75	0.01	0	None	2
78	SR430	2013	WMFS	WMFS	2.75	0.13	4.3	Low	1
79	SR524	2021	Hoima	WMFS	2.73	0	0	None	NA
80	SR518	2021	Hoima	WMFS	2.73	0.04	0	None	4
81	SR333	2013	Oyam	NMFS	2.73	0.07	18	Low	3
82	SR422	2013	Kayunga	LVC	2.7	0.04	0	None	3
83	SR529	2021	Hoima	WMFS	2.7	0.01	0	None	NA
84	SR302	2013	Hoima	WMFS	2.7	0.04	96	medium	1
85	SR525	2021	Hoima	WMFS	2.67	0.06	95.3	Medium	3
86	SR55	2013	Mbale	LVC	2.65	0.17	0	None	1
87	SR531	2021	Bukoba	Tanzania	2.63	0.07	0.7	Low	4
88	SR506	2020	Lwengo	LVC	2.63	0.08	0	None	3
89	SR459	2021	Sironko	LVC	2.6	0.08	13.3	low	3

90	SR528	2021	Hoima	WMFS	2.6	0.04	43	low	5
91	SR48	2013	Luwero	LVC	2.6	0.04	1.01	low	4
92	SR512	2021	Arua	WNFS	2.6	0.01	50	medium	1
93	SR454	2021	Hoima	WMFS	2.57	0.04	0	None	1
94	SR476	2021	Sironko	LVC	2.55	0.03	109.7	High	5
95	SR406	2013	Luwero	LVC	2.54	0.17	29.68	low	5
96	SR41	2013	Kyenjojo	WMFS	2.54	0.03	0	None	1
97	SR406	2013	Luwero	LVC	2.53	0.01	29.68	Low	5
98	SR448	2021	Hoima	WMFS	2.53	0.08	0	None	5
99	SR460	2021	Sironko	LVC	2.52	0	0	None	2
100	SR474	2021	Sironko	LVC	2.52	0	110	medium	5
101	SR471	2021	Sironko	LVC	2.5	0.02	15	Low	3
102	SR70	2013	Mubende	LVC	2.5	0.09	332	High	1
103	SR471	2021	Sironko	LVC	2.5	0.01	15	Low	3
104	SR439	2021	Hoima	WMFS	2.5	0.13	483	High	5
105	SR513	2021	Arua	WNFS	2.5	0.08	0	None	1
106	SR488	2021	Sironko	LVC	2.5	0	1.7	low	3
107	SR35	2013	Kitgum	NMFS	2.5	0.05	65	Medium	3
108	SR439	2021	Hoima	WMFS	2.5	0.05	483	High	5
109	SR70	2013	Mubende	LVC	2.5	0.01	332	High	1
110	SR519	2021	Hoima	WMFS	2.48	0.08	40	Low	5
111	SR514	2021	Arua	WNFS	2.48	0.17	0	None	1
112	SR502	2020	Oyam	NMFS	2.48	0.02	45.3	Low	NA
113	SR207	2013	Jinja	LVC	2.48	0	63	Medium	3
114	SR437	2021	Hoima	WMFS	2.47	0.09	276.7	High	5
115	SR5	2013	Amuria	TFZ	2.45	0.07	20.68	low	1
116	SR497	2020	Oyam	NMFS	2.45	0.01	133.7	low	5
117	SR4	2013	Amuria	TFZ	2.4	0.09	0	None	3
118	SR436	2021	Hoima	WMFS	2.4	0.01	60	Medium	5
119	SR323	2013	WMFS	WMFS	2.4	0.06	37	Low	1
120	SR209	2013	Sironko	LVC	2.38	0.02	85	Medium	3
121	SR287	2013	Mbarara	WMFS	2.37	0.04	235	High	5
122	SR485	2021	Sironko	LVC	2.37	0.07	0	None	1
123	SR2	2013	Amuria	TFZ	2.35	0.04	0	None	1
124	SR515	2021	Hoima	WMFS	2.35	0.02	15	Low	5
125	SR356	2013	Lira	NMFS	2.34	0.03	112	medium	5
126	SR336	2013	Oyam	NMFS	2.34	0.11	32	Low	3
127	SR335	2013	Oyam	NMFS	2.33	0.07	2	low	3
128	SR200	2013	Kapchorwa	EH	2.33	0.05	311	High	5
129	SR335	2013	Oyam	NMFS	2.33	0.04	2	low	3
130	SR332	2013	Oyam	NMFS	2.3	0.05	37	Low	1
131	SR8	2013	Apac	NMFS	2.3	0.05	0	None	1
132	SR30	2013	Kisoro	SWH	2.3	0.09	26.01	low	5
133	SR498	2020	Oyam	NMFS	2.3	0	119	medium	3
134	SR343	2013	Oyam	NMFS	2.3	0.02	2	Low	NA
135	SR444	2021	Hoima	WMFS	2.3	0.05	90	Medium	5
136	SR321	2013	Oyam	NMFS	2.3	0.17	2	Low	3

137	SR334	2013	Oyam	NMFS	2.3	0.13	32	Low	3
138	SR466	2021	Sironko	LVC	2.28	0.04	0	medium	5
139	SR330	2013	Oyam	NMFS	2.25	0.06	58	Medium	5
140	SR141	2013	Wakiso	LVC	2.25	0.01	52	Medium	NA
141	SR454	2021	Hoima	WMFS	2.23	0.05	0	None	1
142	SR45	2013	Luwero	LVC	2.21	0.11	76.01	medium	5
143	SR417	2013	Bukedea	TFZ	2.21	0.08	0	None	3
144	SR22	2013	Kapchorwa	EH	2.2	0.03	366	High	NA
145	SR28	2013	Kabale	SWH	2.2	0.01	8.35	low	5
146	SR23	2013	Kabale	SWH	2.2	0.08	543	High	5
147	SR235	2013	Arua	WNFS	2.17	0.03	21	Low	1
148	SR509	2021	Arua	WNFS	2.15	0	0	None	1
149	SR8	2013	Apac	NMFS	2.15	0.01	0	None	1
150	SR527	2021	Hoima	WMFS	2.12	0.11	0	None	1
151	SR511	2021	Arua	WNFS	2.1	0.08	0	None	1
152	SR402	2013	Kisoro	LVC	2.1	0	0	None	5
153	SR25	2013	Kabale	SWH	2.1	0.07	0	None	4
154	SR87	2021	Mbale	LVC	2.1	0	0	None	NA
155	SR26	2013	Kabale	SWH	2.1	0.09	70.01	medium	2
156	SR492	2021	Sironko	LVC	2.1	0.03	0	None	1
157	SR414	2013	Kabarole	SWH	2.1	0.17	333	High	4
158	SR29	2013	Kabale	SWH	2.1	0.13	60	medium	5
159	SR37	2013	Kyenjojo	WMFS	2.1	0.11	49	Low	5
160	SR325	2013	Oyam	NMFS	2.1	0.04	2	Low	3
161	SR2	2013	Amuria	TFZ	2.1	0.11	0	None	1
162	SR31	2013	Kisoro	SWH	2.07	0.01	33.01	low	5
163	SR31	2013	Kisoro	SWH	2.07	0.02	33.01	low	5
164	SR31	2013	Kisoro	SWH	2.07	0.01	33	low	5
165	SR297	2013	Kabarole	SWH	2.07	0.03	248	High	5
166	SR478	2021	Sironko	LVC	2.05	0.01	0	None	1
167	SR228	2013	Koboko	WNFS	2.05	0.06	58	Medium	1
168	SR510	2021	Arua	WNFS	2.03	0.02	0	None	2
169	SR279	2013	Arua	WNFS	2.03	0.07	49	Low	1
170	SR443	2021	Hoima	WMFS	2.03	0.07	0	None	1
171	SR229	2013	WMFS	WMFS	2	0.08	0	None	NA
172	SR24	2013	Kabale	SWH	2	0.08	33	low	5
173	SR2	2013	Amuria	TFS	2	0.09	0	None	1
174	SR4	2013	Amuria	TFZ	2	0.01	0	None	3
175	SR201	2013	Kapchorwa	EH	1.95	0.17	0	None	5
176	SR523	2021	Hoima	WMFS	1.9	0.06	0	None	1
177	SR59	2013	Mbale	LVC	1.9	0.01	0	None	4
178	SR11	2013	Apac	NMFS	1.9	0.07	0	None	NA
179	SR57	2013	Mbale	LVC	1.9	0	38	low	3
180	SR59	2013	Mbale	LVC	1.9	0.04	0	None	4
181	SR225	2013	Arua	WNFS	1.85	0.05	17	Low	5
182	SR533	2013	Mbale	LVC	1.78	0.03	0	None	NA
183	SR493	2021	Sironko	LVC	1.73	0.07	5	Low	1

184	SR493	2021	Sironko	LVC	1.73	0.09	5	Low	1
185	SR208	2013	Sironko	LVC	1.7	0.09	253	High	5
186	SR33	2013	Kitgum	NMFS	1.5	0.13	42	Low	1
187	SR9	2013	Apac	NMFS	1.25	0.08	0	None	1
188	SR500	2020	Oyam	NMFS	1.05	0.02	0	None	1

EH- Eastern Highlands, LVC- Lake Victoria Crescent and Mbale Farmland, NMFS- Northern Mixed Farming System, SWH- South Western Highlands, TFZ- Teso Farming Zone, WMFS- Western Mixed Farming System, WNFS- West Nile Mixed Farming System.
