# Supplementary material for: Pathogenic and Genetic Diversity of Sclerotium rolfsii, the Causal Agent of Southern Blight of Common Bean in Uganda
Source: J Fungi (Basel). 2025 Dec 26;12(1):18. doi: 10.3390/jof12010018 (PMC12843155; doi:10.3390/jof12010018)
Supplement: Supplementary file 1 [file jof-12-00018-s001.zip › Table S1.pdf]

#**Table S1.** List of *S. rolfsii* strains, their agro-ecology of origin, virulence, and genetic cluster. Strains that have NA under genetic cluster were not present during genotyping

S/no	Strain	Agroecology	District	DSI	SE	Genetic cluster
1	SR282	WNFS	Kabale	93.4	8.6	5
2	SR417	TFZ	Bukedea	91.7	4.2	3
3	SR406	LVC	Luweero	84.5	2.2	5
4	SR70	LVC	Mubende	84.3	7.8	1
5	SR497	NMFS	Oyam	82.2	1.5	5
6	SR52	LVC	Luweero	81.8	1.5	3
7	SR505	LVC	Kamuli	80.9	0.7	5
8	SR59	LVC	Mbale	80.6	2.9	4
9	SR56	LVC	Mbale	80.3	1.5	2
10	SR410	LVC	Mbale	80.1	1.9	5
11	SR523	WMFS	Hoima	80	0.9	1
12	SR208	LVC	Sironko	79.2	7.3	5
13	SR74	LVC	Nakaseke	78.6	1.1	1
14	SR26	SWH	Kabale	78.5	3.4	2
15	SR48	LVC	Luweero	78.3	0.1	4
16	SR37	WMFS	Kyenjojo	78	0	5
17	SR531	TANZANIA	Bukoba	77.7	0.8	4
18	SR439	WMFS	Hoima	77.3	0	5
19	SR35	NMFS	Kitgum	77	2.3	3
20	SR530	TANZANIA	Bukoba	76.5	0	4
21	SR402	SWH	Kisoro	75.8	0.1	5
22	SR6	LVC	Apac	75.2	3.6	5
23	SR51	LVC	Luweero	75.2	3	NA
24	SR28	SWH	Kabale	75.2	0.3	5
25	SR225	WNFS	Arua	75	6.9	5
26	SR78	LVC	Nakaseke	74.7	0	NA
27	SR25	SWH	Kabale	74.5	1.9	4
28	SR335	NMFS	Oyam	74.3	6.9	3
29	SR323	Unknown	Unknown	74.2	6.9	1
30	SR32	LVC	Kamuli	73.9	2	1
32	SR510	WNFS	Arua	72.3	0.9	2
33	SR237	LVC	Lwengo	71.7	1.6	5
34	SR438	WMFS	Hoima	71	3.6	5
35	SR415	LVC	Mukono	70.9	1.1	1
36	SR466	LVC	Sironko	70.8	0.9	5
37	SR471	LVC	Sironko	70.8	3.7	3
38	SR53	LVC	Mbale	70.7	0.1	5
39	SR38	WMFS	Kyenjojo	70.4	2	1
40	SR407	LVC	Luweero	70.4	1.2	3
41	SR425	LVC	Sironko	70.2	0	3
42	SR65	LVC	Mukono	69.8	1.4	1
43	SR46	LVC	Luweero	69.5	0.9	1
44	SR24	SWH	Kabale	69.5	4.8	5

45	SR421	LVC	Luweero	69.1	0.2	3
46	SR77	LVC	Nakaseke	68.8	0.1	4
47	SR434	WMFS	Hoima	68.6	0.6	NA
48	SR11	NMFS	Apac	68.2	4.7	NA
49	SR30	SWH	Kisoro	68	1.6	5
50	SR55	LVC	Mbale	67.9	2.8	1
51	SR15	NMFS	Gulu	67.7	1.1	NA
52	SR436	WMFS	Hoima	67.7	3.7	5
53	SR139	LVC	Sironko	67.3	0	5
54	SR141	LVC	Wakiso	67.2	0.6	NA
55	SR519	WMFS	Hoima	67.1	3.8	5
56	SR12	NMFS	Apac	67	2.5	NA
57	SR279	WNFS	Arua	66.3	1.8	1
58	SR5	TFZ	Amuria	65.2	0	1
59	SR299	WMFS	Ibanda	64.9	6	NA
60	SR201	EH	Kapchorwa	64.8	6	5
61	SR47	LVC	Luweero	64.8	2.6	5
62	SR330	NMFS	Oyam	64.3	6	5
63	SR241	LVC	Masaka	64.3	5.9	NA
64	SR509	WNFS	Arua	63.5	1.6	1
65	SR281	WNFS	Arua	63.4	5.9	5
66	SR23	SWH	Kabale	63.3	0	5
67	SR527	WMFS	Hoima	63.3	3.2	1
68	SR478	LVC	Sironko	63	2.3	1
69	SR296	LVC	Rakai	62.2	5.8	NA
70	SR482	LVC	Sironko	61.9	0	5
71	SR449	WMFS	Hoima	61.7	2.1	4
72	SR14	WNFS	Arua	61.6	0	NA
73	SR27	SWH	Kabale	61.6	0.3	NA
74	SR400	LVC	Mbale	61.4	5.7	5
75	SR356	NMFS	Lira	61.3	5.7	5
76	SR422	LVC	Kayunga	61.2	0.2	3
77	SR339	NMFS	Oyam	61.2	5.7	NA
78	SR413	LVC	Kayunga	60.8	1.1	1
79	SR520	WMFS	Hoima	60.8	0	NA
80	SR244	LVC	Masaka	60	5.6	5
81	SR287	WMFS	Mbarara	59.7	5.5	5
82	SR464	LVC	Sironko	59.7	3.9	2
83	SR514	WNFS	Arua	59.5	0	1
84	SR297	WMFS	Kabarole	59.3	5.5	5
85	SR31	SWH	Kisoro	58.2	0	5
86	SR456	LVC	Sironko	58.1	0	1
87	SR2	TFZ	Amuria	58	0	1
88	SR501	NMFS	Oyam	58	3.7	5
89	SR455	WMFS	Hoima	57.8	0	NA
90	SR487	LVC	Sironko	57.6	1.7	3
91	SR207	LVC	Jinja	57.4	5.3	1

92	SR250	LVC	Rakai	56.4	5.2	1
93	SR332	NMFS	Oyam	56.1	5.2	1
94	SR499	NMFS	Oyam	56.1	0	NA
95	SR477	LVC	Sironko	55.6	0	5
96	SR57	LVC	Mbale	55.6	4	3
97	SR217	NFS	Lira	55.2	5.1	NA
98	SR252	LVC	Masaka	55	5.1	1
99	SR465	LVC	Sironko	54.1	5	3
100	SR461	LVC	Sironko	53.8	1.6	NA
101	SR512	WNFS	Arua	53.5	1.7	1
102	SR522	WMFS	Hoima	53.2	0.4	5
103	SR33	NMFS	Kitgum	53.1	2	1
104	SR432	WMFS	Hoima	52.6	2.4	NA
105	SR481	LVC	Sironko	52.2	2	2
106	SR235	WNFS	Arua	52.1	4.8	1
107	SR431	Unknown	Unknown	51.3	2.8	NA
108	SR468	LVC	Sironko	50.4	0	5
109	SR293	WMFS	Kyenjojo	50.2	4.6	NA
110	SR442	WMFS	Hoima	50.1	0	NA
111	SR508	WNFS	Arua	50	2.3	5
112	SR408	LVC	Nakaseke	50	1.1	4
113	SR445	WMFS	Hoima	49.6	0.4	NA
114	SR462	LVC	Sironko	49.5	6.5	3
115	SR67	LVC	Mukono	49.3	2.8	1
116	SR3	TFZ	Amuria	48.8	7	NA
117	SR203	LVC	Kamuli	48.3	4.5	5
118	SR249	LVC	Masaka	48.1	4.5	5
119	SR504	NMFS	Oyam	47.3	0	5
120	SR290	WNFS	Kamwenge	46.9	4.3	1
121	SR283	WMFS	Kyenjojo	46.4	4.3	1
122	SR443	WMFS	Hoima	45.8	0	1
123	SR302	WMFS	Hoima	45.1	4.2	1
124	SR492	LVC	Sironko	45.1	3.9	1
125	SR240	LVC	Rakai	45	4.2	NA
126	SR437	WMFS	Hoima	45	3	5
127	SR385	Unkbown	Unknown	43.3	3	NA
128	SR29	SWH	Kabale	42.6	3.6	5
129	SR414	WMFS	Kabarole	42.5	0	4
130	SR459	LVC	Sironko	42.3	3.8	3
131	SR511	WNFS	Arua	41.7	5.2	1
132	SR1	TFZ	Amuria	41.4	2.1	1
133	SR336	NMFS	Oyam	41.3	3.8	3
134	SR476	LVC	Sironko	40.3	3.9	5
135	SR8	NMFS	Apac	40.2	5	1
136	SR228	WNFS	Koboko	40.1	3.7	1
137	SR41	LVC	Kayunga	40.1	0	1
138	SR491	LVC	Sironko	39.3	3.9	3

139	SR516	LVC	Sironko	39.3	1.3	NA
140	SR500	NMFS	Oyam	38.5	3.4	1
141	SR63	LVC	Mukono	37.1	0	3
142	SR334	NMFS	Oyam	36.9	3.4	3
143	SR513	WNFS	Arua	36.6	1.6	1
144	SR446	WMFS	Hoima	36.4	0.9	5
145	SR495	LVC	Sironko	36.3	1.3	5
146	SR476	LVC	Sironko	40.3	3.9	5
147	SR8	NMFS	Apac	40.2	5	1
148	SR228	WNFS	Koboko	40.1	3.7	1
149	SR430	WMFS	Hoima	35.9	5.2	1
150	SR411	WMFS	Hoima	35.4	4.2	NA
151	SR333	NMFS	Oyam	35	3.2	3
152	SR521	WMFS	Hoima	34.9	0	NA
153	SR483	LVC	Sironko	33.3	0	NA
154	SR321	NMFS	Oyam	33.2	3.1	3
155	SR325	NMFS	Oyam	33.1	3.1	3
156	SR43	LVC	Luweero	32.8	0	NA
157	SR485	LVC	Sironko	32.3	5.1	1
158	SR209	LVC	Sironko	31.9	3	3
159	SR452	WMFS	Hoima	31.9	0	NA
160	SR433	WMFS	Hoima	31.5	0	5
161	SR474	LVC	Sironko	30.6	1.7	5
162	SR488	LVC	Sironko	29.2	0.6	3
163	SR16	NMFS	Gulu	28.6	3.5	NA
164	SR460	LVC	Sironko	28.5	3.5	2
165	SR4	TFZ	Amuria	28.4	0.5	3
166	SR205	LVC	Jinja	28.3	2.6	3
167	SR444	WMFS	Hoima	27.8	2.9	5
168	SR528	WMFS	Hoima	27.4	1.5	5
169	SR69	LVC	Mubende	26.1	1.7	NA
170	SR200	EH	Kapchorwa	24.7	2.3	5
171	SR494	LVC	Sironko	24.5	3.5	4
172	SR49	LVC	Luweero	24	0.9	NA
173	SR484	LVC	Sironko	23.2	0.5	3
174	SR256	LVC	Masaka	22.9	3.6	2
175	SR525	WMFS	Hoima	22.1	0	3
176	SR515	LVC	Sironko	20.9	1.3	4
177	SR448	WMFS	Hoima	18.3	2.9	5
178	SR498	NMFS	Oyam	18.1	0.3	3
179	SR45	LVC	Luweero	18	2.9	5
180	SR493	LVC	Sironko	16.8	2.1	1
181	SR435	WMFS	Hoima	16.1	0	5
182	SR454	WMFS	Hoima	16	1.9	1
183	SR264	LVC	Bugiri	15.9	1.5	NA
184	SR518	LVC	Sironko	15.5	1.9	4
185	SR506	LVC	Lwengo	13.2	1.5	3

186	SR9	NMFS	Apac	10.2	0.1	1
187	SR489	LVC	Sironko	10.1	1.4	NA
188	SR475	LVC	Sironko	10.1	1.1	2

EH- Eastern Highlands, LVC- Lake Victoria Crescent and Mbale Farmland, NMFS- Northern Mixed Farming System, SWH- South Western Highlands, TFZ- Teso Farming Zone, WMFS- Western Mixed Farming System, WNFS- West Nile Mixed Farming System.
