# Supplementary material for: Pathogenic and Genetic Diversity of Sclerotium rolfsii, the Causal Agent of Southern Blight of Common Bean in Uganda
Source: J Fungi (Basel). 2025 Dec 26;12(1):18. doi: 10.3390/jof12010018 (PMC12843155; doi:10.3390/jof12010018)
Supplement: Supplementary file 1 [file jof-12-00018-s001.zip › Figure S2.pdf]

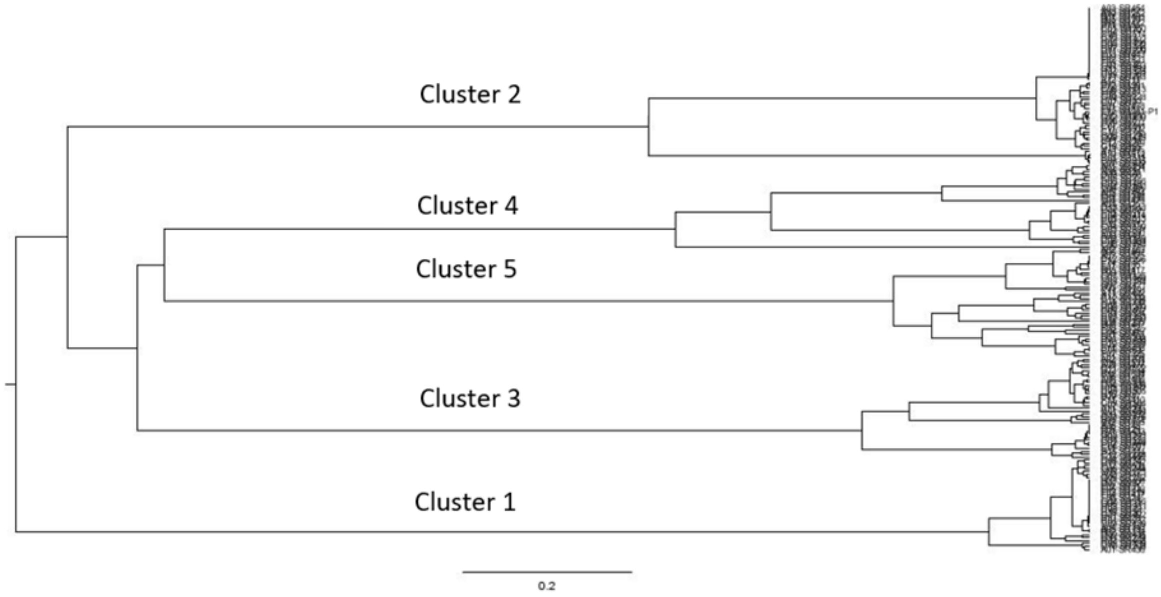

Figure S2. A phylogenetic tree generated using unweighted pair group method with arithmetic mean (UPGMA) based on the two principal components and visualized using FigTree v1.4.4.
