# Supplementary material for: Pathogenic and Genetic Diversity of Sclerotium rolfsii, the Causal Agent of Southern Blight of Common Bean in Uganda
Source: J Fungi (Basel). 2025 Dec 26;12(1):18. doi: 10.3390/jof12010018 (PMC12843155; doi:10.3390/jof12010018)
Supplement: Supplementary file 1 [file jof-12-00018-s001.zip › Table S7.pdf]

Table S7. Pair wise comparison of Wrights fixation Indices between the different genetic clusters

	Cluster 2	Cluster 3	Cluster 4	Cluster 5
**Cluster 0**				
**Cluster 1**	0.671			
**Cluster 2**	0.351	0.416		
**Cluster 3**	0.588	0.385	0.312	
**Cluster 4**	0.778	0.487	0.493	0.670
