# Supplementary material for: Pathogenic and Genetic Diversity of Sclerotium rolfsii, the Causal Agent of Southern Blight of Common Bean in Uganda
Source: J Fungi (Basel). 2025 Dec 26;12(1):18. doi: 10.3390/jof12010018 (PMC12843155; doi:10.3390/jof12010018)
Supplement: Supplementary file 1 [file jof-12-00018-s001.zip › Table S6.pdf]

Table S6. The allele frequency for SNP Chr3_3160729 and SNP Chr3_2484656 among the different genetic clusters

**Genetic cluster**	**SNP Chr3_3160729**		**SNP chr3_2484656**	
	**Chr3_3160729.T**	**chr3_3160729.A**	**chr3_2484656.A**	**chr3_2484656.T**
1	0.94000	0.06000	0.8000	0.2000
2	1.00000	0.00000	1.0000	0.0000
5	1.00000	0.00000	1.0000	0.0000
3	0.09375	0.90625	0.1875	0.8125
4	1.00000	0.00000	1.0000	0.0000
