# Supplementary figures and images for: Pathogenic and Genetic Diversity of Sclerotium rolfsii, the Causal Agent of Southern Blight of Common Bean in Uganda
Source: J Fungi (Basel). 2025 Dec 26;12(1):18. doi: 10.3390/jof12010018 (PMC12843155; doi:10.3390/jof12010018)

**Figure S1.** *S. rolfii* Genomic DNA band pictures and the field ID's. L is the ladder

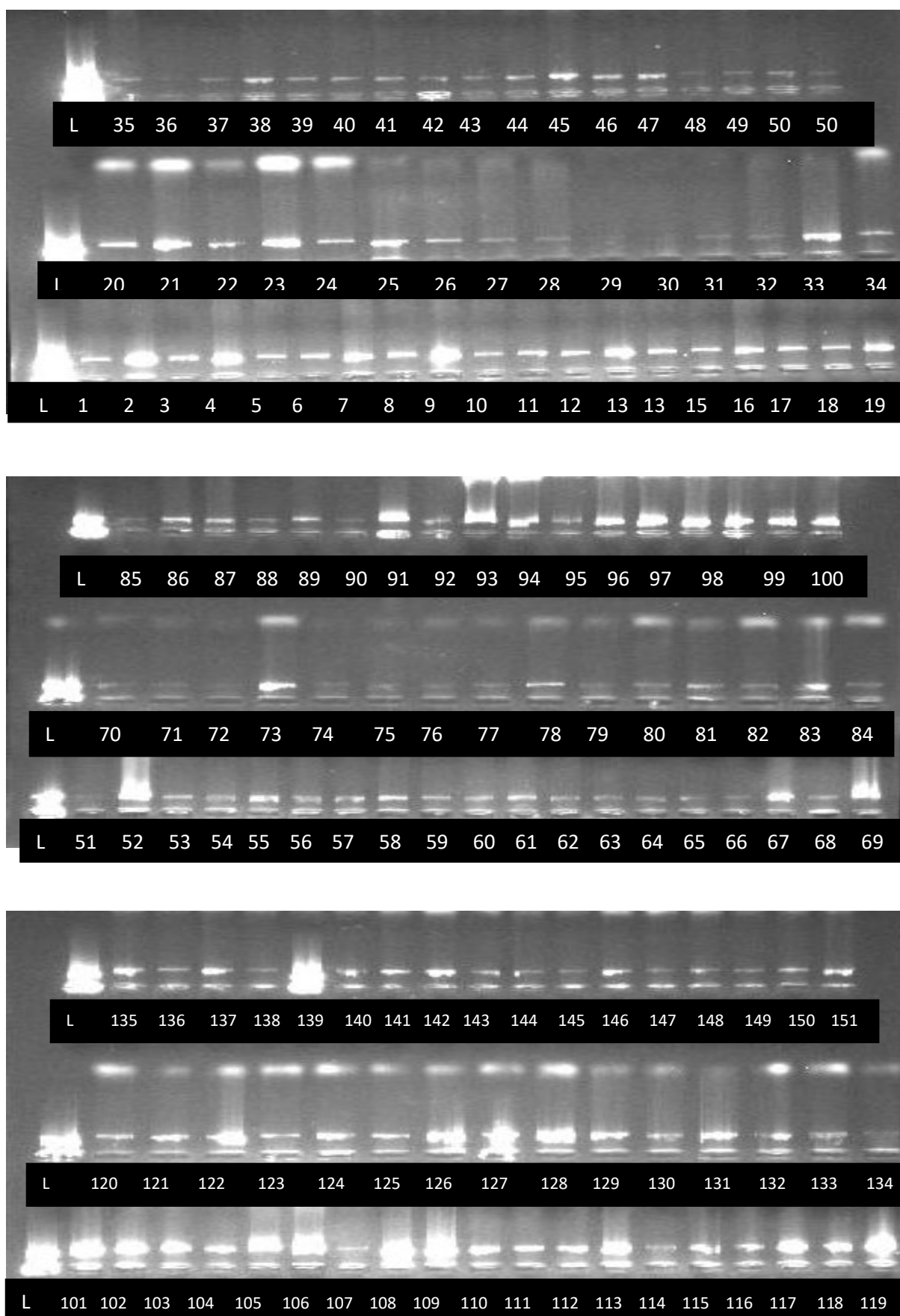

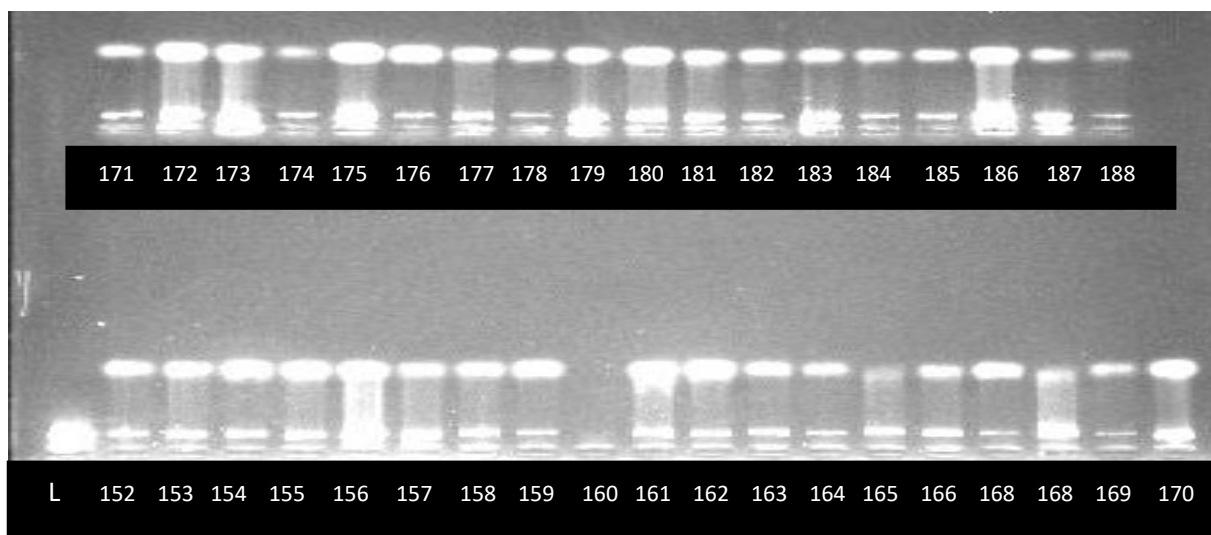

Supplement: Supplementary file 1 [file jof-12-00018-s001.zip › Figure S1.pdf]
